# The role of plant species and soil condition in the structural development of the rhizosphere

**DOI:** 10.1111/pce.13529

**Published:** 2019-03-22

**Authors:** Jon R. Helliwell, Craig J. Sturrock, Anthony J. Miller, W. Richard Whalley, Sacha J. Mooney

**Affiliations:** ^1^ Division of Agricultural and Environmental Sciences, Gateway Building, Sutton Bonington Campus University of Nottingham Leicestershire LE12 5RD UK; ^2^ Metabolic Biology, John Innes Centre Norwich Research Park Norwich NR4 7UH UK; ^3^ Sustainable Soils and Grassland Systems Department Rothamsted Research, West Common, Harpenden Hertfordshire AL5 2JQ UK

**Keywords:** rhizosphere, root diameter, soil structure, structural development, X‐ray computed tomography (CT)

## Abstract

Roots naturally exert axial and radial pressures during growth, which alter the structural arrangement of soil at the root–soil interface. However, empirical models suggest soil densification, which can have negative impacts on water and nutrient uptake, occurs at the immediate root surface with decreasing distance from the root. Here, we spatially map structural gradients in the soil surrounding roots using non‐invasive imaging, to ascertain the role of root growth in early stage formation of soil structure. X‐ray computed tomography provided a means not only to visualize a root system in situ and in 3‐D but also to assess the precise root‐induced alterations to soil structure close to, and at selected distances away from the root–soil interface. We spatially quantified the changes in soil structure generated by three common but contrasting plant species (pea, tomato, and wheat) under different soil texture and compaction treatments. Across the three plant types, significant increases in porosity at the immediate root surface were found in both clay loam and loamy sand soils and not soil densification, the currently assumed norm. Densification of the soil was recorded, at some distance away from the root, dependent on soil texture and plant type. There was a significant soil texture × bulk density × plant species interaction for the root convex hull, a measure of the extent to which root systems explore the soil, which suggested pea and wheat grew better in the clay soil when at a high bulk density, compared with tomato, which preferred lower bulk density soils. These results, only revealed by high resolution non‐destructive imagery, show that although the root penetration mechanisms can lead to soil densification (which could have a negative impact on growth), the immediate root–soil interface is actually a zone of high porosity, which is very important for several key rhizosphere processes occurring at this scale including water and nutrient uptake and gaseous diffusion.

## Abstract 2

By high resolution imagery, we observed across the three plant types significant increases in porosity at the immediate root surface, irrespective of soil texture and bulk density, and not soil densification, the currently assumed norm. This has profound implication for several key rhizosphere processes occurring at this scale including water and nutrient uptake and gaseous diffusion that should be considered in plant breeding efforts.

## 1 INTRODUCTION

The dynamic nature of the rhizosphere (the zone of soil surrounding a growing root, which is influenced by it) provides a niche environment, which exhibits biophysical and chemical gradients very different to those found away from the soil immediately influenced by the root, referred to as the bulk soil. These gradients control root activity through a combination of root‐derived exudations and physical structural alterations, influencing water and nutrient uptake, gaseous exchange, particle rearrangement, and wettability at the immediate root surface. Carminati et al. (2010) revealed the influence of mucilage on the water holding capacity of the soil immediately around the root, and its implications for hydraulic continuity around the root system was demonstrated by (Moradi et al., 2011). In compacted soils, the influence of plant derived exudates have been highlighted to improve mechanical conditions for root penetration (Oleghe, Naveed, Baggs, & Hallett, 2017). Carminati and Vetterlein (2013) proposed the concept of rhizosphere plasticity to help understand the bimodal hydraulic responses found at the root–soil interface under different bulk soil moisture conditions. However, soil structural dynamics, particularly around an actively growing root, have been largely limited to theoretical models (Dexter, 1988) or root analogue approaches (Aravena, Berli, Ghezzehei, & Tyler, 2011) due to the inherent difficulties in observing a fragile, opaque system in situ.

As impeded roots elongate, they undergo radial and axial elongations (Misra, Dexter, & Alston, 1986), exerting compressive and shear forces on the surrounding soil in horizontal and vertical directions (Bengough & MacKenzie, 1994; Kolb, Hartmann, & Genet, 2012). It is known that root diameter varies in response to compaction and soil strength, with many studies demonstrating an increased radial expansion of the root axes in dense soil (Atwell, 1988; Materechera, Dexter, & Alston, 1991; Tracy et al., 2012). These pressures, generated by the root, are partly responsible for soil structural alterations in the rhizosphere, and they in turn affect the hydraulic continuity of the pore system (Aravena et al., 2011). However, the exact effect of root growth on soil structure, especially at the scale of the pore, is uncertain, in large part due to the limited number of studies, which have compared root responses under contrasting physical soil conditions for different plant species (Iijima & Kato, 2007; Materechera, Dexter, & Alston, 1992). Aravena et al. (2011) reported decreased porosity around growing roots using a root analogue technique, which showed the radial forces in wet soil reduce interaggregate pore space, impacting on the hydraulic contact between aggregates. Contrary to this, Helliwell et al. (2017) recently reported an increase in porosity at the immediate root surface at a resolution of 12 μm, surrounding the growing roots of tomato in both coarse and fine soil textures, with a decrease in porosity observed away from the root in the bulk soil.

The functioning of the rhizosphere and, in particular, its role in regulating the hydraulic behaviour of plants have been active areas of research for many years. Carminati et al. (2013) showed the importance of gap formation around roots in decreasing transpirational demand in lupin. Likewise, Berli, Carminati, Ghezzehei, and Or (2008) highlighted the potentially beneficial role of rhizosphere densification in increasing hydraulic contact and connectivity between neighbouring aggregates. Hence, understanding how plants influence the precise arrangement of soil around a root in terms of densification, gap formation, and the resulting impact on water and nutrient flow towards roots is very important from a plant developmental perspective. Ascertaining the role of root growth on the structure of the rhizosphere is challenging due to the fragile nature of soils. Previous attempts to address this have employed thin‐section microscopy through resin impregnation, to “fix” and preserve the root and soil systems prior to analysis (Mooney, Morris, Craigon, & Berry, 2007; Veen, Vannoordwijk, Dewilligen, Boone, & Kooistra, 1992). However, these techniques are very laborious, still allow for substantial root and soil disturbance, and do not readily enable the study of the system in 3‐D. Non‐invasive imaging such as X‐ray computed tomography (CT), X‐ray radiography, neutron radiography, and magnetic resonance imaging are now accepted methods that are assisting us overcoming these limitations having been successfully employed in studies of plant–soil interactions over the last decade (see reviews by Helliwell et al., 2013; Mooney, Pridmore, Helliwell, & Bennett, 2012; Pires, Borges, Bacchi, & Reichardt, 2010; Taina, Heck, & Elliot, 2008). Recent advances in X‐ray detector efficiencies, X‐ray source power, and image analysis methodologies have also highlighted X‐ray CT as an exciting tool for mapping microscale alterations to root architectures and soil structures (Helliwell et al., 2013; Mooney et al., 2012), with previous limitations of coarse resolutions and poor image quality greatly reduced.

The objective of this study was to take advantage of the recent advances in imaging methodology to visualize the root‐mediated soil structure in 3D (e.g., Helliwell et al. 2017) and gain a new insight into root‐induced physical transformations in the rhizosphere. The first aim was to assess how three different plant species with contrasting root architecture modify the soil structure at the immediate soil surface in comparison with the bulk soil. Second, we sought to investigate how the root response to the soil was influenced by soil texture (or particle size) as this has often been ignored in previous studies that have tended to focus on one soil type. Finally, we examined the root response to soil structuring when grown in soils at different bulk densities to assess the impact of compaction. Based on previous work, we hypothesized that while root growth mechanisms would generate zones of higher soil density, the root–soil interface, a key zone for water and nutrient exchange, would be a zone of higher porosity consistent across all species.

## 2 MATERIALS AND METHODS

### 2.1 Soil core preparation and sampling

Four replicate columns (80‐mm height × 25‐mm diameter) per soil texture and per bulk density were uniformly packed to 1.2 and 1.5 Mg m^−3^ with air‐dried sieved (<2 mm) Newport series loamy sand (sand 83.2%, silt 4.7%, and clay 12.1%; pH 6.35; organic matter 2.93%; Food and Agriculture Organization (FAO) (United Nations) brown soil) and Worcester series clay loam (sand 35.6%, silt 31.5%, and clay 32.9%; pH 6.50; organic matter 5.19%; FAO Argillic Pelosol) soil from the University of Nottingham farm at Bunny (Nottinghamshire, UK—52.52°N, 1.07°W). The water retention curves for these soils can be found in Helliwell, Miller, Whalley, Mooney, and Sturrock (2014). To ensure homogeneity in sample preparation and reduce any effects of soil slumping following packing into the cores, the samples underwent one wetting and drying cycle using tension table apparatus, before being maintained at a tension of −5 kPa on the tension table throughout seedling establishment and growth. Previous work in Helliwell et al. (2017) showed that this was optimal for soil structure stabilization without inducing noticeable cracking through shrinkage. Surgical micropore tape (3M United Kingdom PLC, Bracknell) was placed over the columns during soil preparation to reduce soil surface evaporation and prevent sample contamination, while still enabling gaseous exchange. Seeds of tomato Solanum lycopersicum *cv*. “Ailsa Craig,” winter wheat Triticum aestivum *cv*. “Cordiale,” and common pea Pisum sativum *cv*. “Kelvedon Wonder” were germinated in the dark on wetted filter paper for 48 hr before being planted 5 mm below the soil surface in the replicate columns for each soil texture and bulk density combination (*n* = 48). Plants were grown under controlled conditions (22°C day/16°C night); 40% relative humidity; a 12‐hr photoperiod with a photosynthetic photon flux density at plant level of 330 μmol m^−2^ s^−1^ in a climate chamber for a period of 8 days. During this 8‐day period, the plants are mainly using nutrient seed reserves to support growth (Bouaziz & Hicks, 1990), and there was insufficient time for the development of nitrogen‐fixing nodules on the pea roots.

### 2.2 X‐ray CT scanning procedure

The samples were scanned using two X‐ray microtomography systems, in order to assess plant‐induced structural development across two different spatial resolutions. All samples were initially scanned using a Phoenix Nanotom 180NF X‐ray micro‐CT scanner (GE Sensing and Inspection Technologies, Wunstorf, Germany). The source had a 3‐μm focal spot, with the centre of the sample 5.4 cm from the X‐ray source and a resultant imaged voxel size of 12 μm. The entire sample was imaged with a field of view of 2,308 × 2,308 pixels using an X‐ray energy of 110 kV, a current of 110 μA and an exposure time of 750 ms. A 0.2‐cm Cu filter was used, and 1,600 image projections were taken, with each scan taking 70 min to complete. Each sample was scanned once 8 days after planting, exposing each plant to a calculated dose of 6.33 Gy (Zappala et al., 2013).

A subsection of two replicates per plant × soil texture × soil bulk density treatment were further scanned using a Phoenix v|tome|x m 240 kV X‐ray micro‐CT scanner (GE Sensing and Inspection Technologies, Wunstorf, Germany). Due to an improved detector efficiency (allowing enhanced X‐ray projection image collection) and higher X‐ray flux in this system, scans at a voxel spatial resolution of 8.5 μm were possible, with each scan taking 43 min to complete. An X‐ray energy of 120 kV and current of 60 μA was used, with 1,981 projections taken at a timing of 333 ms per projection. The centre of the sample was 3.48 cm from the X‐ray source. Each sample was scanned once, also after 8 days, exposing each plant to a calculated dose of 7.52 Gy.

### 2.3 Image processing, segmentation, and analysis

Image processing was performed in VG StudioMax® 2.2 software, using procedures largely detailed in Helliwell et al. (2017). Briefly, segmentation of soil, root, and pore phases was undertaken after applying a median filter of Radius 3 pixels to remove noise but preserve structural borders. To segment pore and soil phases, the greyscale histogram was calibrated (individually for each sample) against pore space and a common aluminium reference object, segmenting solid material from pore and organic (including root) material. At this high resolution and early growth stage, the roots were readily segmented using an adaptive region growing algorithm, starting from the greyscale value of the user‐selected voxel and selecting all connected voxels within the user defined range. The entire segmented root architecture from this point was analysed as a whole. To assess changes to soil structure with distance from the root surface, the surface mesh of the root region was three‐dimensionally (3‐D) dilated, creating discreet regions moving away from the root in which pore and soil volumes could be calculated. The first one‐voxel dilation was subtracted from all subsequent dilations to prevent any mischaracterization at the immediate root surface due to partial volume effects or noise. The “Volume Analyser” tool was used to assess the volume of pore and soil material within each dilated region, giving porosity profiles (where one voxel = 12 μm) for each zone moving away from the root surface. This could be compared with a bulk soil value, taken as the porosity of a large volume of soil observed at the furthest distance away from the root, but without being influenced by the container wall (i.e., in most cases, c. 1 cm from the edge). The short growth period of the experiment meant that roots did not interact with the boundary of the container; however, to minimize any potential impact of this, we excluded material from the edges (c. 2 mm) from the analysis. By this method, we analysed the full root system of each plant. No roots overlapped for the imaging undertaken at 8.5 μm; however, for the wheat plants scanned at 12 μm, two samples had instances of roots in close proximity or overlapping, which were excluded from the study; however, as extra samples had been prepared and scanned, *n* = 4 for each treatment was maintained.

Root diameter was assessed by the novel application of an existing image analysis protocol. A binary image stack of thresholded root material was exported from the VG Studio Max v2.2 volume and imported into Image J 1.47 (http://rsbweb.nih.gov/ij/). Here, 3‐D thickness measurements were made on root systems using the BoneJ plugin (Doube et al., 2010). This plugin places sequentially smaller spheres inside the object of interest, and each sphere never overlaps the object border or each other. The mean diameter of these spheres is deemed the “thickness,” giving a single value for each root system. A subsequent colour heat map can be used to illustrate changes to relative sphere size to give an indication of soil pore thickness change along the root axis.

Root convex hull can be used to provide a measure of potential soil exploration by different plant root systems (Iyer‐Pascuzzi et al., 2010), by assigning straight vertices between the outer most points of the root system. Convex hull was determined by importing the segmented root systems into *RooTrak* software (Mairhofer et al., 2013) using the QuickHull algorithm (Barber, Dobkin, & Huhdanpaa, 1996) and estimating hull volume using Monte Carlo Integration (Rubinstein, 1981).

### 2.4 Statistical analysis

All data were analysed in GenStat Release 15.1 (VSN International) using a single‐variate linear mixed model restricted maximum likelihood (REML), containing all possible interactions as explanatory variables and sample as a random effect. For soil porosity analysis, a REML analysis containing plant species, soil texture, the distance from the root surface, and soil bulk density as the fixed model and sample as a random effect was used. Standard residual plots were examined in GenStat to check data normality, with comparisons of means based on least significant differences at the *P* = 0.05 and *P* = 0.01 levels.

## 3 RESULTS

### 3.1 The influence of root growth on rhizosphere porosity

**Figure 1:**
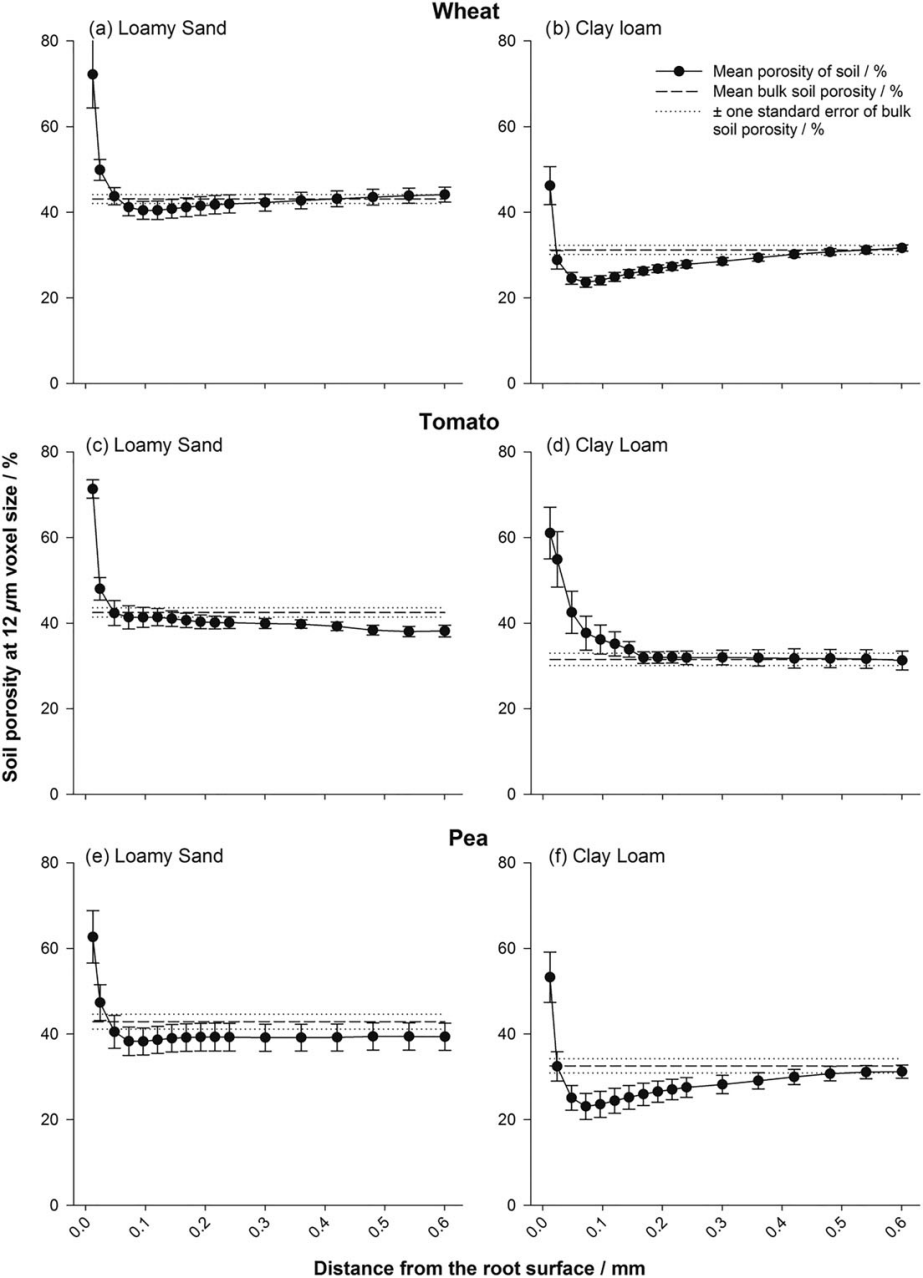
Porosity distributions at a bulk density of 1.2 Mg m^−3^ for wheat (a,b), tomato (c,d), and pea (e,f) roots: (a,c,e) loamy sand and (b,d,f) clay loam soils, at isolated regions moving away from the root surface. Error bars represent standard errors of four replicates

**Figure 2:**
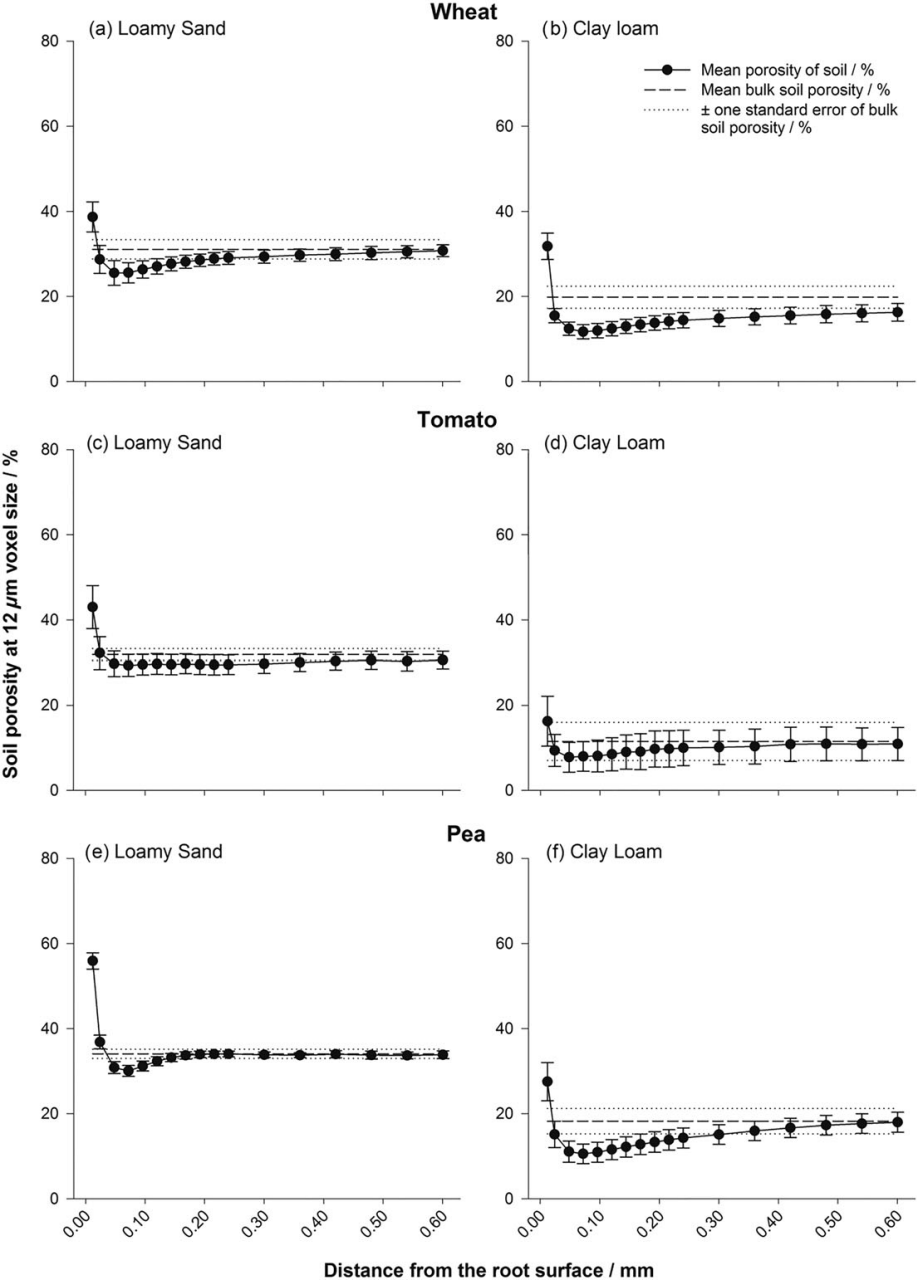
Porosity distributions at a bulk density of 1.5 Mg m^−3^ for wheat (a,b), tomato (c,d), and pea (e,f) roots: (a,c,e) loamy sand and (b,d,f) clay loam soils, at isolated regions moving away from the root surface. Error bars represent standard errors of four replicates

There was a clear gradient in porosity surrounding the root systems in all treatments after 8 days of growth (Figures 1 and 2), with an enhanced porous zone at the immediate root surface in all samples and treatment specific localized compaction/densification at increased distance from the root. “Densification” was considered as the point at which the porosity of an individual dilated region became statistically the same or lower than that of the bulk soil. The interaction of bulk density × plant species × soil texture × distance from the root surface was significant (*P* < 0.001).

**Figure 3:**
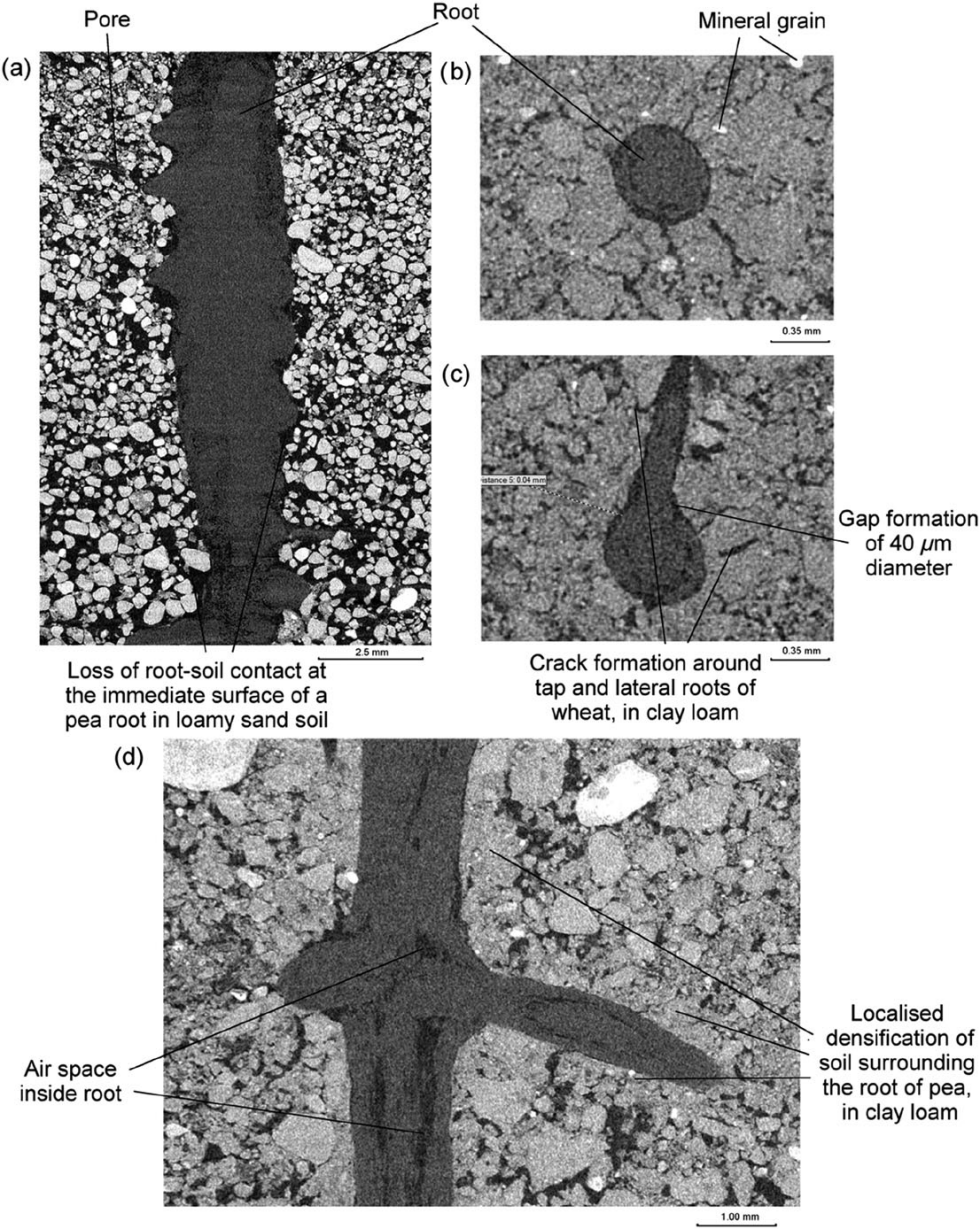
Representative raw greyscale X‐ray CT images showing soil, root, and pore space after 8 days of growth: (a) Pea in loamy sand soil showing gap formation immediately at the root surface; (b,c) wheat in clay loam soil showing cracks radiating from the root surface; and (d) pea in clay loam soil showing densification of the soil surrounding the root

When averaged over all treatments, there was a significant increase in soil porosity at the immediate root surface compared with 48 μm away from the root (mean porosity of 47.3% and 26.8% respectively; Figures 1 and 2; *P* < 0.001; SE's available in Tables S1–S4), with a significant interaction for plant species × distance from the root (*P* < 0.05), soil texture × distance from the root (*P* < 0.001), and bulk density × distance from the root (*P* < 0.001). Scanning at a higher resolution revealed a clear gap formation around tap and lateral roots in both soil textures (Figure 3), the diameter of which approximately equalled the zones of increased porosity quantified in Figures 1 and 2. Beyond this initial gap formation, changes to porosity at increased distance from the root surface were explained by soil texture and bulk density.

At a bulk density of 1.2 Mg m^−3^, the loamy sand soil exhibited no further significant change to porosity compared with the bulk soil at increasing distances away from the root surface for any plant species (Figure 1a,c,e). At the same bulk density in the clay loam, there was no significant change in porosity from the bulk soil value for the tomato treatment (Figure 1d), but significant reductions in porosity of 7.5 and 9.5% compared with the bulk soil value to 23.6% and 23.1% in the wheat and pea species, respectively (Figure 1b,f; *P* < 0.001). This localized densification compared with the bulk soil extended to 0.36 and 0.42 mm from the root surface in the wheat and pea species, respectively, with the soil particularly compressed at the 0.1 mm location for both species compared with the root–soil interface.

At 1.5 Mg m^−3^, the tomato plants exhibited no further changes in porosity following the initial increase at the immediate root surface in either soil texture (Figure 2c,d), although the differences in the soil porosity profile between the two textures were the most pronounced observed. However, there were significant decreases in porosity in wheat and pea plants in both soil textures (Figure 2a,b,e,f; *P* < 0.001), the magnitude of which were texture specific. In the loamy sand, the wheat and pea plants exhibited decreases in porosity compared with the bulk soil of 5.6% and 4.0%, respectively, with localized soil densification extending to 0.14 and 0.12 mm from the root surface. In the clay loam, the wheat and pea plants exhibited greater decreases in porosity of 8.1% and 7.6%, respectively, compared with the bulk soil than in the loamy sand. Densification of the soil surrounding the root extended further than in the loamy sand, to 0.42 and 0.22 mm from the root surface in the clay loam for the wheat and pea treatments, respectively.

The zone of influence of the root (i.e., the spatial degree of any change in porosity away from the bulk soil) as an isolated dependent variable was significantly influenced by plant species (*P* < 0.001) in the following the order wheat > pea > tomato (means of 694.7, 483.9, and 21.2 mm^3^, respectively). Soil texture also significantly influenced the zone of influence (*P* < 0.05), with clay loam having a much higher volume of 511.7 mm^3^ compared with 288.2 mm^3^ in the loamy sand. The bulk density × texture interaction was significant (*P* = 0.05), with a larger zone of influence in the clay at 1.5 than 1.2 Mg m^−3^ (mean values of 630.3 and 402.9 mm^3^, respectively), but in sand, it was the converse (mean values of 225.2 and 334.5 mm^3^, respectively). In comparison with the lower density soil, the porosity at the root–soil interface and the bulk soil was reduced by between 25% and 50% in the 1.5 Mg m^−3^ of treatment.

### 3.2 Impact of soil physical properties on root characteristics

**Figure 4:**
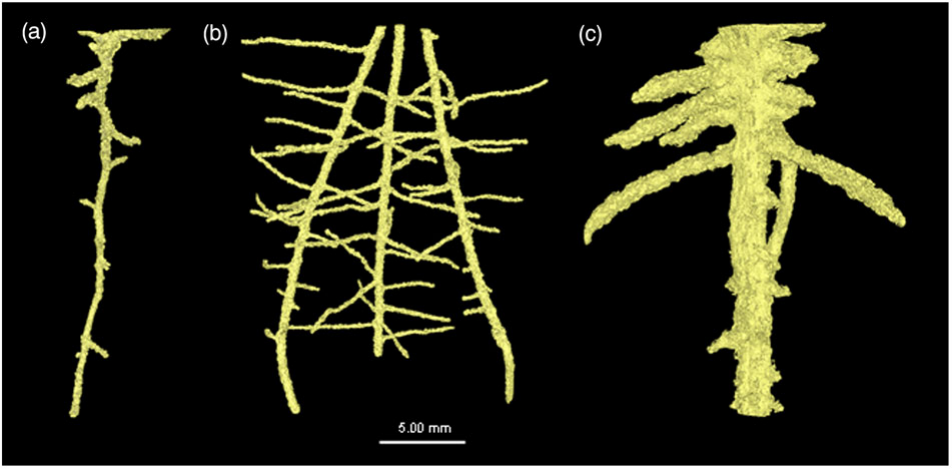
Example root system architectures at a bulk density of 1.5 Mg m^−3^ for (a) tomato, (b) wheat, and (c) pea [Colour figure can be viewed at wileyonlinelibrary.com]

**Figure 5:**
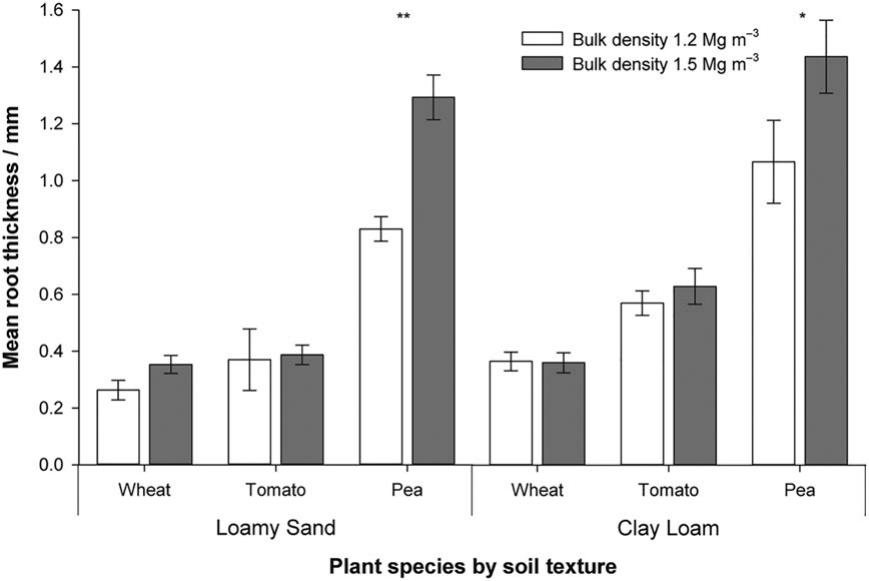
The influence of bulk density and plant species on root thickness after 8 days of growth. Error bars represent standard errors of four replicates. Significance: ^*^ *P* < 0.05 and ^**^ *P* < 0.01

Representative images of root system architecture segmented from the X‐ray CT images for the three plant species are provided in Figure 4. Mean root thickness increased with increasing bulk density (0.58 mm vs. 0.74 mm at bulk densities of 1.2 and 1.5 Mg m^−3^, respectively; *P* = 0.001), with a significant interaction of bulk density × plant species (Figure 5; *P* < 0.005). Root thickness significantly differed between plant species with the following the order: pea > tomato > wheat (mean thickness values of 1.16, 0.49 and 0.34 mm, respectively; Figure 5; *P* < 0.001). Root thickness varied significantly with soil type (*P* < 0.001), increasing in the finer textured clay loam (mean thickness of 0.74 vs. 0.58 mm in the clay loam and loamy sand textures, respectively). The interaction of species × texture was significant (*P* = 0.01). Averaged across all treatments, there was no significant effect of root thickness on porosity of the defined rhizosphere region, but a significant interaction of plant species × root zone of influence (*P* < 0.005) and bulk density × plant species × root zone of influence (*P* = 0.001). Note, this is based on analysis of the soil around the roots hence where root architecture varied so did the volume of soil assessed.

**Figure 6:**
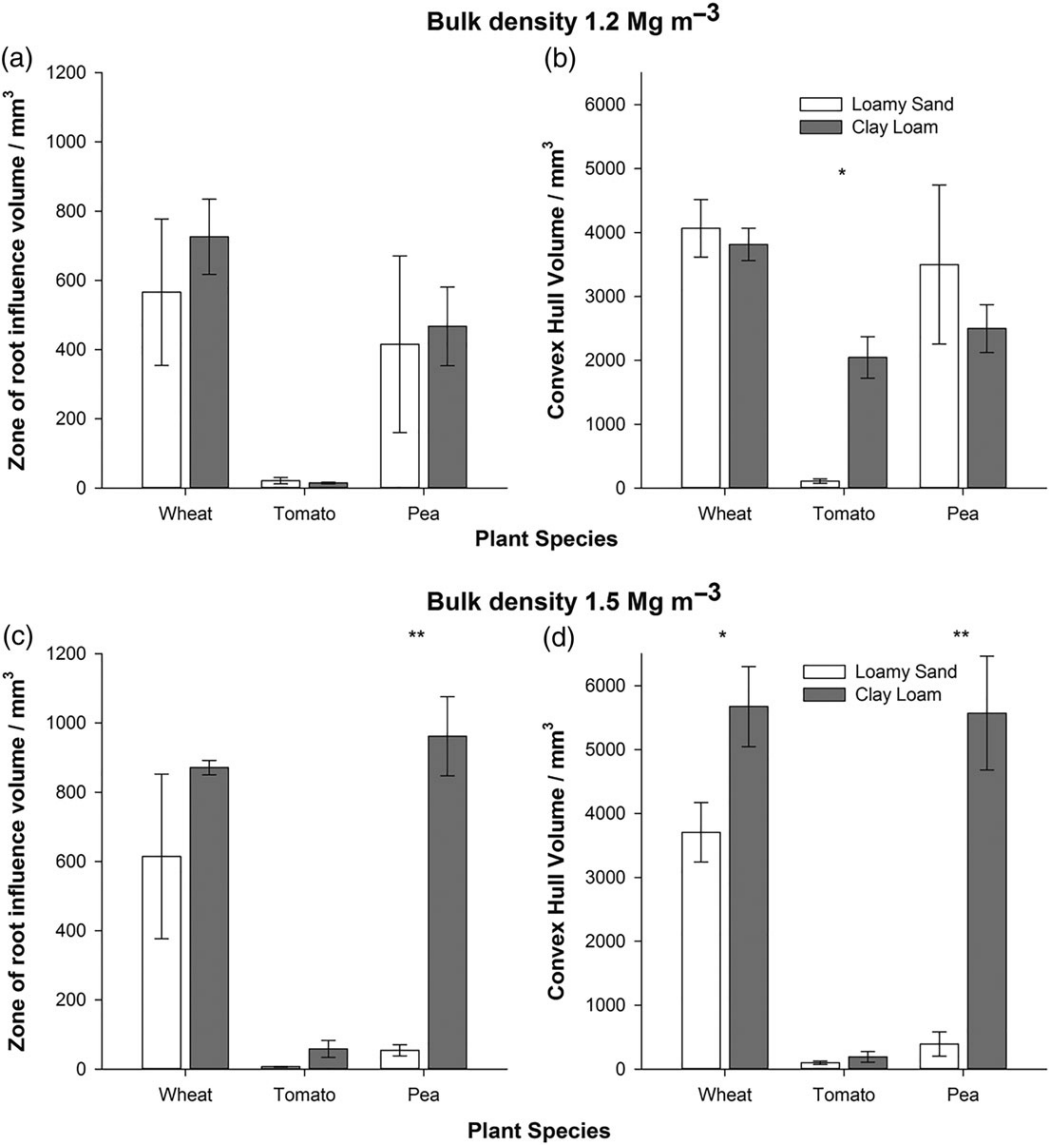
The influence of plant species and soil type on (a) mean root zone of influence at 1.2 Mg m^−3^; (b) convex hull volume for 1.2 Mg m^−3^; (c) mean root zone of influence at 1.5 Mg m^−3^; and (d) convex hull volume for 1.5 Mg m^−3^. Error bars associated with the histograms represent standard errors of four replicates. Significance: ^*^ *P* < 0.05 and ^**^ *P* < 0.001

Mean values for convex hull volume were higher in the clay loam than loamy sand (5,607 vs. 4,060 mm^3^; Figure 6; *P* < 0.005) and were significantly affected by plant species (convex hull volumes of 7,077, 3,940, and 3,483 mm^3^ in the wheat, pea, and tomato, respectively; *P* < 0.001). There were significant interactions of bulk density × soil texture (*P* < 0.05) and bulk density × species × texture (*P* < 0.05). There was a significant relationship between convex hull volume and the volume of the root zone of influence (*P* < 0.001), with mean total volumes of both convex hull and the volume of root zone of influence differing dramatically between plant species (*P* < 0.001) and soil texture (*P* < 0.005; Figure 6).

**Figure 7:**
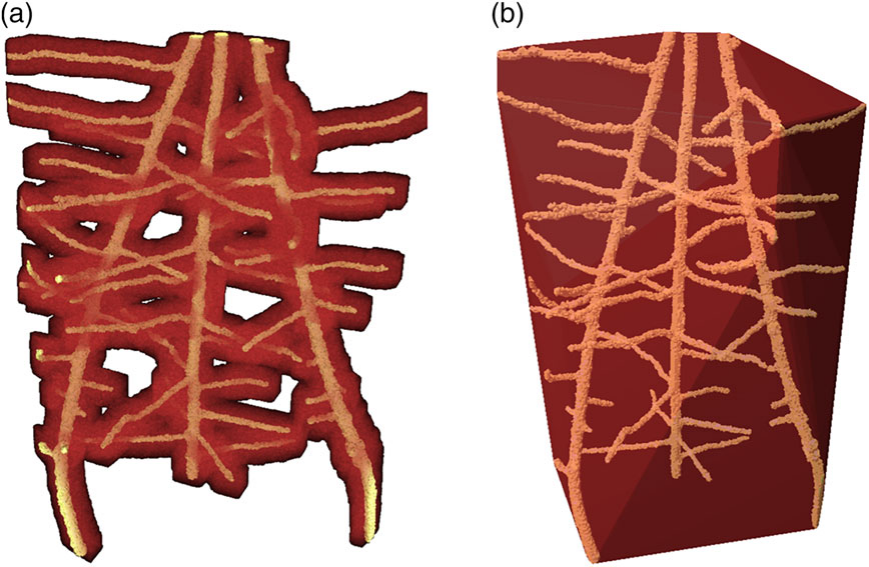
An example of the (a) volume of the zone of influence of the root and (b) convex hull for wheat: with the segmented root system in yellow and associated zone of influence in red

## 4 DISCUSSION

Root growth has a significant impact on soil structure in the rhizosphere, which we observed here after very early growth. The extent of soil reorganization is influenced not only by the plant but also by the soil's physical properties. Previous work has indicated that soil structure in the rhizosphere has key consequences for soil physical (Gregory, 2006; Hinsinger, Bengough, Vetterlein, & Young, 2009) and hydraulic processes that directly influence root system development (Carminati et al., 2013; Hallett et al., 2009). Although previous work, such as Aravena et al. (2011), used root analogues to try disentangle the consequences of root growth on structural development in the rhizosphere, an assessment of real growing roots in field soil on rhizosphere structure evolution has previously been considered not possible. In this study, we used X‐ray CT to observe the structural development of the rhizosphere across multiple plant species and soil treatments at scales down to 8.5 μm on soil from the field that was structure less. This approach offer new opportunities to study in situ how plants influence the soil environment to their advantage/disadvantage and how this is affected by different abiotic stresses.

### 4.1 Impact of root growth on rhizosphere porous architecture

There was a plant species independent increase in porosity immediately at the root surface, which subsequently declined with distance from the root previously measured by Helliwell et al. (2017). This contrasts with the previous work using root analogues (Aravena et al., 2011), which demonstrated a soil densification gradient at the immediate root surface, increasing in porosity with distance from the root. Aravena et al. (2011) acknowledge limitations to their balloon root analogue, in that, it consists of an unreactive nondynamic interface, isolating lateral compressive forces due to radial expansion. Therefore, in a real root system, more dynamic differences in the structural gradients from the root to the bulk soil are expected. Beyond this zone of increased porosity, an increase in densification of the soil was observed, governed by soil texture (Figures 1 and 2). Figure 3b,c highlights the development of cracking behaviour in the clay loam soil, with root‐derived cracks radiating from the root surface in all plant species. This is almost certainly due to shrinkage induced by soil drying (Hallett & Newson, 2005) and not a sample preparation artefact because great care was taken to ensure the samples were packed as homogenously as possible following the method of Helliwell et al. (2017). The plastic nature of the clay loam can lead to the formation of localized microcracks during root growth, corresponding to and accounting for the increases in porosity quantified at the immediate root surface (Figure 1). The loamy sand texture, which has a much smaller capacity to shrink than clay loam, exhibited a smaller but measurable shrinkage upon drying at the root surface, linked to a loss of contact, which was particularly pronounced in the thicker pea roots (Figure 3a). However, as this soil did not crack, the magnitude of porosity increase, estimated from the CT images, was smaller (Figure 2).

New lateral root growth was observed in crack shaped pores in the soil, with an apparent preference for growth into pre‐existing pore space as opposed to forging new pathways. New root proliferation is known to exploit existing pore channels and fissures where possible (Bengough et al., 2006), due to the relatively unimpeded pathways in these regions compared with denser surrounding soil, although the extent of this can be regulated by the overall soil bulk density (Colombi, Braun, Keller, & Walter, 2017). Hence, root growth often becomes clustered in these channels that bypass stronger regions of the soil (White & Kirkegaard, 2010), creating hotspots of intense water and nutrient uptake and zones of relatively unaffected soil in poorly explored impenetrable areas (Passioura, 2002). It is likely that the increased yield observed in some zero tillage systems is due to enhanced root penetration at depth due to an increased frequency of biopores and enhanced pore connectivity (Pittelkow et al., 2015). Roots can also proliferate to locally exploit patches of nutrients (Drew, 1975). However, as the soil was homogenized before packing into columns in this investigation, we can discount root exploitation of pre‐existing nutrient patches. We observed that roots exhibited a clear strategy where lateral roots explore newly formed fissures, potentially as an energy conservation mechanism. This also accounts for a degree of gap formation immediately around the tap and lateral roots (Figure 3b,c), as the roots often failed to fully fill the pores. The importance of gap formation around growing roots was highlighted by Carminati et al. (2013), with the shrinkage of roots responsible for air‐filled gaps particularly pronounced around the tap root. However, Carminati et al. (2013) and other previous investigations (Carminati, Vetterlein, Weller, Vogel, & Oswald, 2009) demonstrated shrinkage of the root as opposed to the soil was the driver for the gap development dynamics. It is possible that shrinkage of the soil was overlooked in previous work due to the coarser resolution (*~*100 μm); thus, microscale structural changes were not observed. Also, the high sand content (92%) used by Carminati et al. (2013) would limit shrinkage of the soil itself, a likely factor influencing rhizosphere structure development. The role of root hairs in structural formation is not considered here due to an inability to observe them in these soils at the prescribed moisture content (due to an overlap in X‐ray attenuation rather than resolution), although Koebernick et al. (2017) has shown this is possible in a coarse textured soil via synchrotron imaging when considering air‐filled pores only.

Beyond the initial increase in porosity at the immediate root surface, the contrasting porosity changes at distances further from the root surface are also likely to be influenced by the different cohesive properties of the soil. It follows that an apparent lack of densification surrounding roots growing in coarser, less cohesive soil is due to its relative ductility, with freely mobile particles able to be reorganized as the root grows. Conversely, the plastic nature of the clay soil creates a readily compressible mass, clearly influenced by root size. The root effect on increasing densification away from the root interface was greater in the highest bulk density treatment and was consistent between the two soil types, though Figure 5 shows that this cannot be explained by root diameter alone.

We hypothesized a relationship between the thickness of a root, soil bulk density, and the degree and size of its impact on the surrounding physical soil environment, with thicker roots under increased soil bulk density thought to contribute to an increased deformation of rhizosphere soil. The nonsignificant effect of root thickness as a factor determining the “zone of influence” shows that root diameter, although reported to increase the ability of roots to penetrate compacted soil, (Bengough, 1997), does not account for the changes to structure we have observed once the rhizosphere has developed. The combination of thicker (Figure 4) and blunter pea roots under the appropriate soil texture exhibited increased soil deformation compared with tomato and wheat (Figures 1f and 2f). Although the degree of structural change was independent of root thickness, the displacement of particles was less than one root diameter in all treatments. This contrasts with Vollsnes, Futsaether, and Bengough (2010) who showed compression of sand in front of the root tip extending up to eight times the root diameter in maize using particle image velocimetry. Aravena et al. (2011) reported lateral densification of *~*8–12% extending to one root diameter in wet aggregates at a resolution of 4.4 μm. In this investigation, we observed a similar degree of deformation of *~*4–9% depending on the soil texture and plant species, extending *~*0.5× the root diameter (although root‐induced cracking often extended beyond this; Figure 4b,c). It is therefore clear that investigations using artificial sand or a saturated medium may cause differences in the size and magnitude of structural change observed not representative of field soil conditions.

A common feature we observed was that immediately adjacent to the root; there was a region of increased porosity. This was most likely due some combination of both soil and root shrinkage alongside the thigmotropic response of root development. The way in which particles, especially in structure‐less samples, are arranged at the root–soil interface has been proposed to account for the zone of higher porosity (Koebernick et al., 2018), and although we cannot discount that this as a contributing factor, it is clear from Figure 3b–d where a particulate structure is not observed, that this is unlikely to explain our findings. At greater distances from the root, there was a compacted region (except for Figure 1d), which was due to either (a) a legacy of soil deformation at the root tip or (b) microscale soil shrinkage due to water uptake by the root. Differences in root exudate composition between the plant species are also thought to be important in modifying the physical properties soil (Naveed et al., 2017).

### 4.2 Implications for modelling of rhizosphere densification

Dexter (1987) developed a model for the compression of soil surrounding a growing root by assuming soil porosity is reduced adjacent to the root where compression is greatest. This was based on work considering a metal probe as a root analogue entering the soil and expanding to cause a porosity gradient, which increased exponentially from the object surface (Dexter & Tanner, 1972). This was later supported by experimental work using particle image velocimetry in pure sand at a spatial resolution of 0.5 mm (Vollsnes et al., 2010), where the displacement of sand particles into pores in their immediate vicinity was facilitated by root growth. Our work confirmed the predictions by Dexter (1987) that following root compression of soil to a minimum porosity and an example of this behaviour is seen in Figure 3d. However, we more commonly observed a dual‐zone impact of root growth on soil structure in the rhizosphere (Figures 1 and 2), with the first corresponding to the increase in porosity at the immediate root surface to an approximate distance of 50 μm, only observable by high resolution imaging and not previously considered in similar modelling approaches. This high porosity zone where root–soil contact is somewhat reduced could have profound implications for soil root interaction: reduced hydraulic conductivity and water flow to the root due to a loss of hydraulic connection, lower nutrient flux to the root especially nitrate, and increased aeration. The improved aeration could be of considerable benefit to the root whereas the effects related to reduced water flux might be compensated by root mucilage production (Carminati et al., 2009).

Plant roots donate carbon to encourage the development of beneficial populations of microbes in the rhizosphere. For example, phosphate‐solubilizing microorganisms can mobilize previously inaccessible pools of this important nutrient for plants (Wang, Shi, Jiang, Zhang, & Feng, 2016). Microorganisms growing on the root surface contribute to the disruption of soil structure at the root surface that can aid aeration and the pathway for nutrient and water delivery to the root surface (Helliwell et al., 2014). Our finding that the extent of this root surface phenomenon, the zone of influence, differs between species and depends on soil type and density (Figures 6 and 7) is worthy of further investigation. For example, pea showed more sensitivity to the soil type when compared with wheat and tomato at higher bulk density (Figures 6c,d). In the thicker pea roots (Figure 5), the production of specialized exudates particularly rich in hydroxyproline‐rich cell wall glycoprotein when compared with cereals (Knee et al., 2001) may be depend on soil type. There may be the potential to improve this trait in future crop breeding programmes by manipulating root exudate composition. In addition, the considerable differences in root‐induced structure around and away from the root surface and the varied response to soil texture and bulk density highlight the needs for plants breeders to undertake studies under more natural conditions when screening for beneficial root traits.

## 5 CONCLUSIONS

Plants modify the soil environment in the rhizosphere very early on during plant root growth. Soils with contrasting textures are deformed by roots in different ways, depending on initial soil bulk density and plant species. X‐ray microtomography of loamy sand and clay loam soils showed an increase in the porosity of soil immediately adjacent to the root in all three plant species examined, which was independent of root diameter. Multiscale scanning at higher resolutions revealed considerable microcrack formation around roots, attributable to soil shrinkage. However, subsequent deformation and compaction created by root growth was spatially highly heterogeneous, and dependent on a combination of root thickness, higher soil bulk density, and finer textured soils. Imaging approaches, such as those demonstrated here, could provide a basis for the future development of conceptual root–soil interaction models, especially important as the soil structure in the rhizosphere has implications for the acquisition of water and nutrients by plant roots as they engineer new hydraulic pathways through soils. In addition, they could be used to support the efforts of plant breeders when seeking to identify idealized root traits as the root‐modulated soil porous architecture is likely to play as an important role in root development as the root system itself.

## Supporting information

Table S1. Raw porosity data for clay loam samples at a soil bulk density of 1.2 Mg m^‐3^
Table S2. Raw porosity data for loamy sand samples at a soil bulk density of 1.2 Mg m^‐3^
Table S3. Raw porosity data for clay loam samples at a soil bulk density of 1.5 Mg m^‐3^
Table S4. Raw porosity data for loamy sand samples at a soil bulk density of 1.5 Mg m^‐3^
