# Supplementary material for: The role of plant species and soil condition in the structural development of the rhizosphere
Source: Plant Cell Environ. 2019 Mar 22;42(6):1974–86. doi: 10.1111/pce.13529 (PMC6563464; doi:10.1111/pce.13529)
Supplement: Supplementary file 1 — Table S1. Raw porosity data for clay loam samples at a soil bulk density of 1.2 Mg m‐3 Table S2. Raw porosity data for loamy sand samples at a soil bulk density of 1.2 Mg m‐3 Table S3. Raw porosity data for clay loam samples at a soil bulk density of 1.5 Mg m‐3 Table S4. Raw porosity data for loamy sand samples at a soil bulk density of 1.5 Mg m‐3 [file PCE-42-1974-s001.docx]

| **Plant** | **Distance from the root surface / mm** | **Mean Porosity of region / %** | **Standard error for regional porosity** |
| --- | --- | --- | --- |
| Winter Wheat | 0.012 | 46.22 | 4.46 |
|  | 0.024 | 28.86 | 2.14 |
|  | 0.048 | 24.56 | 1.39 |
|  | 0.072 | 23.65 | 1.17 |
|  | 0.096 | 24.11 | 1.08 |
|  | 0.120 | 24.89 | 1.04 |
|  | 0.144 | 25.65 | 0.97 |
|  | 0.168 | 26.26 | 0.93 |
|  | 0.192 | 26.80 | 0.89 |
|  | 0.216 | 27.33 | 0.86 |
|  | 0.240 | 27.85 | 0.84 |
|  | 0.300 | 28.54 | 0.83 |
|  | 0.360 | 29.39 | 0.81 |
|  | 0.420 | 30.20 | 0.77 |
|  | 0.480 | 30.73 | 0.78 |
|  | 0.540 | 31.20 | 0.77 |
|  | 0.601 | 31.65 | 0.73 |
| Pea | 0.012 | 53.26 | 5.86 |
|  | 0.024 | 32.44 | 3.42 |
|  | 0.048 | 25.10 | 2.90 |
|  | 0.072 | 23.11 | 3.03 |
|  | 0.096 | 23.58 | 3.04 |
|  | 0.120 | 24.42 | 2.92 |
|  | 0.144 | 25.21 | 2.75 |
|  | 0.168 | 25.93 | 2.61 |
|  | 0.192 | 26.56 | 2.49 |
|  | 0.216 | 27.06 | 2.40 |
|  | 0.240 | 27.53 | 2.32 |
|  | 0.300 | 28.21 | 2.17 |
|  | 0.360 | 29.08 | 1.88 |
|  | 0.420 | 29.98 | 1.76 |
|  | 0.480 | 30.75 | 1.67 |
|  | 0.540 | 31.11 | 1.54 |
|  | 0.601 | 31.21 | 1.53 |
| Tomato | 0.012 | 61.05 | 6.05 |
|  | 0.024 | 54.90 | 6.47 |
|  | 0.048 | 42.54 | 4.92 |
|  | 0.072 | 37.69 | 3.97 |
|  | 0.096 | 36.19 | 3.41 |
|  | 0.120 | 35.17 | 2.80 |
|  | 0.144 | 33.89 | 1.82 |
|  | 0.168 | 31.94 | 1.38 |
|  | 0.192 | 31.96 | 1.37 |
|  | 0.216 | 32.04 | 1.39 |
|  | 0.240 | 31.90 | 1.59 |
|  | 0.300 | 31.98 | 1.73 |
|  | 0.360 | 31.88 | 1.94 |
|  | 0.420 | 31.73 | 2.27 |
|  | 0.480 | 31.72 | 2.15 |
|  | 0.540 | 31.62 | 2.19 |
|  | 0.601 | 31.26 | 2.22 |

Supplementary Table 1 – Raw porosity data for clay loam samples at a soil bulk density of 1.2 Mg m^-3^

Supplementary Table 2 – Raw porosity data for loamy sand samples at a soil bulk density of 1.2 Mg m^-3^

| **Plant** | **Distance from the root surface / mm** | **Mean Porosity of region / %** | **Standard error for regional porosity** |
| --- | --- | --- | --- |
| Winter Wheat | 0.012 | 72.18 | 7.84 |
|  | 0.024 | 49.88 | 2.44 |
|  | 0.048 | 43.75 | 2.01 |
|  | 0.072 | 41.16 | 2.01 |
|  | 0.096 | 40.43 | 2.09 |
|  | 0.120 | 40.46 | 2.13 |
|  | 0.144 | 40.82 | 2.16 |
|  | 0.168 | 41.16 | 2.19 |
|  | 0.192 | 41.49 | 2.18 |
|  | 0.216 | 41.76 | 2.15 |
|  | 0.240 | 41.96 | 2.10 |
|  | 0.300 | 42.26 | 2.00 |
|  | 0.360 | 42.74 | 1.93 |
|  | 0.420 | 43.15 | 1.84 |
|  | 0.480 | 43.53 | 1.85 |
|  | 0.540 | 43.88 | 1.78 |
|  | 0.601 | 44.11 | 1.76 |
| Pea | 0.012 | 62.67 | 6.13 |
|  | 0.024 | 47.34 | 4.17 |
|  | 0.048 | 40.53 | 3.82 |
|  | 0.072 | 38.31 | 3.30 |
|  | 0.096 | 38.26 | 3.14 |
|  | 0.120 | 38.64 | 3.13 |
|  | 0.144 | 39.00 | 3.18 |
|  | 0.168 | 39.19 | 3.22 |
|  | 0.192 | 39.29 | 3.24 |
|  | 0.216 | 39.32 | 3.26 |
|  | 0.240 | 39.26 | 3.25 |
|  | 0.300 | 39.15 | 3.16 |
|  | 0.360 | 39.15 | 3.12 |
|  | 0.420 | 39.21 | 3.16 |
|  | 0.480 | 39.44 | 3.19 |
|  | 0.540 | 39.43 | 3.19 |
|  | 0.601 | 39.38 | 3.18 |
| Tomato | 0.012 | 71.35 | 2.15 |
|  | 0.024 | 48.02 | 2.64 |
|  | 0.048 | 42.37 | 2.90 |
|  | 0.072 | 41.37 | 2.73 |
|  | 0.096 | 41.36 | 2.33 |
|  | 0.120 | 41.40 | 2.01 |
|  | 0.144 | 41.07 | 1.81 |
|  | 0.168 | 40.68 | 1.68 |
|  | 0.192 | 40.31 | 1.58 |
|  | 0.216 | 40.14 | 1.50 |
|  | 0.240 | 40.14 | 1.37 |
|  | 0.300 | 39.92 | 1.15 |
|  | 0.360 | 39.80 | 0.97 |
|  | 0.420 | 39.27 | 1.03 |
|  | 0.480 | 38.38 | 1.17 |
|  | 0.540 | 38.06 | 1.22 |
|  | 0.601 | 38.18 | 1.37 |

Supplementary Table 3 – Raw porosity data for clay loam samples at a soil bulk density of 1.5 Mg m^-3^

| **Plant** | **Distance from the root surface / mm** | **Mean Porosity of region / %** | **Standard error for regional porosity** |
| --- | --- | --- | --- |
| Winter Wheat | 0.012 | 31.80 | 3.09 |
|  | 0.024 | 15.49 | 1.69 |
|  | 0.048 | 12.42 | 1.56 |
|  | 0.072 | 11.71 | 1.65 |
|  | 0.096 | 11.97 | 1.69 |
|  | 0.120 | 12.45 | 1.68 |
|  | 0.144 | 12.97 | 1.68 |
|  | 0.168 | 13.39 | 1.68 |
|  | 0.192 | 13.77 | 1.68 |
|  | 0.216 | 14.16 | 1.75 |
|  | 0.240 | 14.43 | 1.78 |
|  | 0.300 | 14.83 | 1.86 |
|  | 0.360 | 15.22 | 1.91 |
|  | 0.420 | 15.52 | 1.97 |
|  | 0.480 | 15.83 | 2.02 |
|  | 0.540 | 16.05 | 2.00 |
|  | 0.601 | 16.29 | 2.06 |
| Pea | 0.012 | 27.54 | 4.50 |
|  | 0.024 | 15.13 | 3.08 |
|  | 0.048 | 11.10 | 2.49 |
|  | 0.072 | 10.58 | 2.30 |
|  | 0.096 | 10.96 | 2.34 |
|  | 0.120 | 11.59 | 2.36 |
|  | 0.144 | 12.21 | 2.38 |
|  | 0.168 | 12.80 | 2.40 |
|  | 0.192 | 13.35 | 2.40 |
|  | 0.216 | 13.85 | 2.38 |
|  | 0.240 | 14.32 | 2.36 |
|  | 0.300 | 15.09 | 2.30 |
|  | 0.360 | 15.96 | 2.26 |
|  | 0.420 | 16.68 | 2.28 |
|  | 0.480 | 17.31 | 2.29 |
|  | 0.540 | 17.68 | 2.30 |
|  | 0.601 | 18.00 | 2.35 |
| Tomato | 0.012 | 16.25 | 5.88 |
|  | 0.024 | 9.37 | 3.74 |
|  | 0.048 | 7.80 | 3.49 |
|  | 0.072 | 7.97 | 3.48 |
|  | 0.096 | 8.09 | 3.73 |
|  | 0.120 | 8.49 | 3.88 |
|  | 0.144 | 9.00 | 4.01 |
|  | 0.168 | 9.09 | 4.23 |
|  | 0.192 | 9.71 | 4.25 |
|  | 0.216 | 9.75 | 4.29 |
|  | 0.240 | 9.99 | 4.14 |
|  | 0.300 | 10.11 | 4.01 |
|  | 0.360 | 10.32 | 4.10 |
|  | 0.420 | 10.83 | 4.03 |
|  | 0.480 | 10.92 | 3.97 |
|  | 0.540 | 10.84 | 3.86 |
|  | 0.601 | 10.91 | 3.87 |

Supplementary Table 4 – Raw porosity data for loamy sand samples at a soil bulk density of 1.5 Mg m^-3^

| **Plant** | **Distance from the root surface / mm** | **Mean Porosity of region / %** | **Standard error for regional porosity** |
| --- | --- | --- | --- |
| Winter Wheat | 0.012 | 38.69 | 3.54 |
|  | 0.024 | 28.70 | 3.27 |
|  | 0.048 | 25.53 | 2.89 |
|  | 0.072 | 25.58 | 2.40 |
|  | 0.096 | 26.35 | 2.04 |
|  | 0.120 | 27.06 | 1.81 |
|  | 0.144 | 27.68 | 1.65 |
|  | 0.168 | 28.16 | 1.53 |
|  | 0.192 | 28.53 | 1.46 |
|  | 0.216 | 28.85 | 1.45 |
|  | 0.240 | 29.08 | 1.52 |
|  | 0.300 | 29.36 | 1.52 |
|  | 0.360 | 29.72 | 1.46 |
|  | 0.420 | 29.95 | 1.51 |
|  | 0.480 | 30.24 | 1.56 |
|  | 0.540 | 30.53 | 1.42 |
|  | 0.601 | 30.75 | 1.38 |
| Pea | 0.012 | 55.90 | 1.93 |
|  | 0.024 | 36.83 | 1.61 |
|  | 0.048 | 30.84 | 1.40 |
|  | 0.072 | 30.06 | 1.29 |
|  | 0.096 | 31.20 | 1.17 |
|  | 0.120 | 32.33 | 1.09 |
|  | 0.144 | 33.19 | 0.97 |
|  | 0.168 | 33.71 | 0.85 |
|  | 0.192 | 33.93 | 0.75 |
|  | 0.216 | 34.02 | 0.68 |
|  | 0.240 | 34.04 | 0.67 |
|  | 0.300 | 33.85 | 0.65 |
|  | 0.360 | 33.74 | 0.56 |
|  | 0.420 | 33.98 | 0.85 |
|  | 0.480 | 33.77 | 0.94 |
|  | 0.540 | 33.69 | 0.79 |
|  | 0.601 | 33.85 | 0.88 |
| Tomato | 0.012 | 43.00 | 5.04 |
|  | 0.024 | 32.20 | 3.84 |
|  | 0.048 | 29.70 | 3.01 |
|  | 0.072 | 29.34 | 2.56 |
|  | 0.096 | 29.52 | 2.44 |
|  | 0.120 | 29.64 | 2.41 |
|  | 0.144 | 29.51 | 2.39 |
|  | 0.168 | 29.71 | 2.31 |
|  | 0.192 | 29.51 | 2.30 |
|  | 0.216 | 29.46 | 2.35 |
|  | 0.240 | 29.48 | 2.28 |
|  | 0.300 | 29.69 | 2.24 |
|  | 0.360 | 30.00 | 2.08 |
|  | 0.420 | 30.34 | 2.07 |
|  | 0.480 | 30.54 | 2.10 |
|  | 0.540 | 30.29 | 2.25 |
|  | 0.601 | 30.58 | 2.08 |
